# In Silico Discovery of a Novel Natural Product Targeting PI3Kα for the Treatment of Head and Neck Squamous Cell Carcinoma

**DOI:** 10.3390/ijms26083565

**Published:** 2025-04-10

**Authors:** Wenqing Jia, Xianchao Cheng

**Affiliations:** 1 College of Chemistry and Chemical Engineering, Qilu Normal University, Jinan 250200, China; 2 Tianjin Key Laboratory on Technologies Enabling Development of Clinical Therapeutics and Diagnostics (Theranostics), School of Pharmacy, Tianjin Medical University, Tianjin 300070, China

**Keywords:** ATP competitive PI3Kα inhibitor, natural product, HNSCC, virtual database screening

## Abstract

Head and neck squamous cell carcinoma (HNSCC) remains a major health burden, with abnormal activation of phosphatidylinositol 3-kinase alpha (PI3Kα) strongly implicated in its pathogenesis. Targeting PI3Kα represents a promising therapeutic strategy. In this study, we employed structure-based virtual screening to identify natural small-molecule inhibitors of PI3Kα. A total of 12,800 molecules were screened, and five compounds were selected for further evaluation based on binding affinity and interaction patterns. Pharmacokinetic properties were assessed using ADMET predictions, and molecular dynamics (MD) simulations were conducted to validate the binding stability. Among the candidates, Apigetrin demonstrated favorable ADMET properties, a high safety profile, and stable binding within the ATP-binding pocket of PI3Kα. These findings suggest that Apigetrin is a promising natural PI3Kα inhibitor with potential therapeutic relevance for HNSCC.

## 1. Introduction

Cancer is one of the leading causes of death globally, according to the World Health Organization [1]. Head and neck cancer (HNC) is one of the most common malignant tumors, including oral cancer, nasopharyngeal cancer, laryngeal cancer, hypopharyngeal cancer, and thyroid cancer [2]. Among them, head and neck squamous cell carcinoma (HNSCC) accounts for more than 90% and is defined as a group of malignancies originating from the mucosal epithelium of the oral cavity, pharynx, and larynx [3]. HNSCC has a high incidence rate, ranking as the sixth most common cancer worldwide, with approximately 890,000 new cases diagnosed annually and 450,000 deaths [3]. Despite therapeutic advances, the five-year survival rate for HNSCC remains below 50%, highlighting the urgent need for more effective treatment strategies [3]. HNSCC often disrupts patients’ normal physiological functions, such as breathing, swallowing, and language abilities, consequently leading to a decline in their quality of life [4].

HNSCC can be treated through various methods, including surgery, radiotherapy, chemotherapy, targeted therapy, and immunotherapy. The traditional treatment approach involves a combination of surgery, radiotherapy, and chemotherapy [4]. Surgical intervention poses significant challenges and may adversely affect the patient’s appearance, speech, and swallowing function [5]. Radiotherapy and chemotherapy can control disease progression in the short term [6]. For early-stage (I–II) cases, surgical resection combined with postoperative radiotherapy can yield a more favorable prognosis. However, approximately 60% of patients are already diagnosed with locally advanced disease (III–IV stage) at initial medical treatment. Even with multimodal treatments, like surgery, radiotherapy, and chemotherapy, the risk of local recurrence and distant metastasis remains high [7]. The new approaches in the treatment of HNSCC primarily focus on the refinement of comprehensive treatment methods, as well as the application of novel therapeutic models, such as immunotherapy and targeted therapy. Molecular targeted drugs, which act on specific targets related to tumor development and progression, selectively kill tumor cells while reducing toxic side effects [4].

The therapeutic targets associated with HNSCC include PI3Kα, EGFR, and PD-1/PD-L1 [8]. PI3Kα is a member of the lipid kinases family and regulates proliferation, migration, and invasion, as well as angiogenesis [9]. It is a complex composed of a p85α regulatory and p110α catalytic subunit. The p110α catalytic subunit is encoded by *PIK3CA* [10,11,12]. The PI3K/Akt/mTOR signaling pathway is one of the most classic signaling pathways involving PI3Kα, which is closely related to tumor occurrence and development [13]. The overactivation of the PI3K/Akt/mTOR pathway can be attributed to several factors, including the activation of PI3Kα, the activation of receptor tyrosine kinases (RTKs), and the loss of PTEN function [14]. TCGA statistical results showed that 97 out of 530 HNSCC patients had mutations of *PIK3CA* [4]. Mutations in *PIK3CA*, which result in the abnormal activation of the PI3K pathway, represent one of the most frequent mechanisms driving carcinogenesis [4]. Compared with wild-type PI3Kα, the mutations of *PIK3CA* are functionally acquired mutations, and approximately 80% of mutated sites of *PIK3CA* occur in E542, E545 (helix domain), and H1047 (kinase domain), resulting in unregulated cell growth, proliferation, and survival [15,16,17]. These mutations are detected in approximately 30% of human cancers. They are prevalent in several common cancer types, including endometrial (28%), breast (29%), head and neck (14%), and cervical (17%) cancers [4]. Many RTKs activate PI3K. Upstream RTKs EGFR, ErbB3, Met, and VEGFR, as well as GPCRs, activate PI3K after binding growth factor ligands, promoting the occurrence and development of HNC, cervical cancer, pancreatic cancer, etc. [18,19]. PTEN is a crucial tumor suppressor, and loss of PTEN activity leads to the stable activation of the PI3K/Akt signaling, with subsequent abnormal cell growth, survival, and proliferation [20]. The loss of PTEN is not only common in HNSCC [21] but is also observed in various cancers, such as breast cancer [22] and prostate cancer [23]. Overactivation of the PI3K pathway in HNSCC may occur through *PIK3CA* mutations, RTK activation, and PTEN loss. PI3Kα inhibitors can effectively inhibit the PI3K/Akt/mTOR pathway and exert anti-HNSCC effects. Therefore, PI3Kα is considered a potential target for HNSCC therapy.

**Figure 1:**
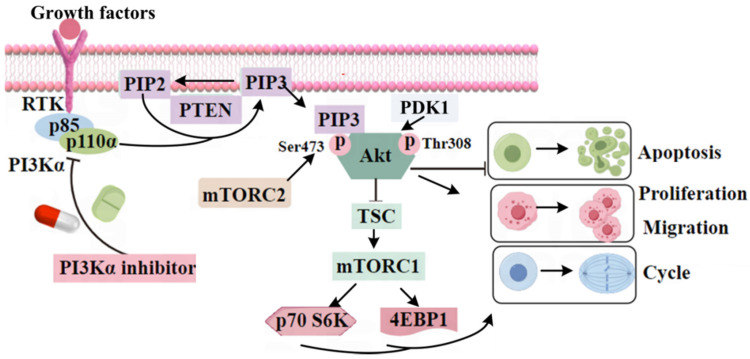
Schematic diagram of the PI3K/Akt/mTOR signaling pathway by Figdraw.

After receiving activation signals, PI3K phosphorylates phosphatidylinositol-4,5-diphosphate (PIP2) to phosphatidylinositol-3,4,5-triphosphate (PIP3), which regulates the downstream signaling pathways [24]. The pleckstrin homology (PH) domain of Akt binds to PIP3 in the plasma membrane. Meanwhile, the conformation of Akt is changed, resulting in its exposure to Thr308 and Ser473. The phosphorylation of Akt at Thr308 by PDK1 and at Ser473 by mTOR complex 2 (mTORC2) results in the full activation of this enzyme [25]. After complete activation, Akt leaves the cell membrane, enters the cytoplasm or nucleus, and activates mTORC1 by phosphorylating the two negative regulatory factors (TSC2 and PRAS40) of mTORC1, further phosphorylating p70S6K and 4EBP1 and regulating cell proliferation and metabolism (Figure 1) [26,27].

Currently, only two PI3Kα inhibitors (alpelisib and inavolisib) have been approved by the FDA, both of which are synthetic drugs. The vast majority of synthetic drugs failed to meet the requirements in clinical trials, such as high toxicity, and were subsequently discontinued. Alpelisib is an oral selective PI3Kα inhibitor, showing significantly improved objective response rates in patients with solid tumors harboring *PIK3CA* mutations (14.2% of HNSCC patients) than those with wild-type tumors. The disease control rate (DCR) in HNSCC patients reached 68.4% (NCT01219699) [28]. Multiple clinical studies have demonstrated that HNSCC patients with *PIK3CA* mutations can benefit from Alpelisib (NCT02282371, NCT03292250, NCT03601507, and NCT02145312). Alpelisib exhibits significant inhibitory effects on both the wild-type and all mutant forms of PI3Kα, but it affects glucose uptake, leading to metabolic dysfunction and even causing hyperglycemia, thereby limiting its clinical application [29]. Inavolisib is a potent PI3Kα inhibitor and mutant PI3Kα degrader used in patients with locally advanced or metastatic *PIK3CA*-mutated solid tumors (NCT03006172) [30]. During the treatment process, specific adverse reactions (AEs), such as hyperglycemia, stomatitis, and rashes, often occur. In addition, CYH33 is a highly selective oral PI3Kα inhibitor, with an objective response rate (ORR) of 14.3% in treating *PIK3CA*-mutated solid tumors, including HNSCC (NCT03544905) [31]. CYH33 promotes the infiltration and activation of CD8+ T cells in tumor tissues, reduces the infiltration of immunosuppressive M2 macrophages, and induces the formation of immune memory [32]. The most frequently reported grade 3/4 treatment-related adverse events include hyperglycemia, rash, decreased platelet count, peripheral edema, and fatigue [31]. Most known PI3Kα inhibitors are synthetic drugs, and their side effects, particularly hyperglycemia, have severely limited the widespread clinical application of this class of drugs.

Natural products have been a valuable and useful source of anticancer drugs. Natural drugs refer to substances naturally produced by organisms, such as plants, animals, insects, aquatic organisms, and microorganisms, which possess pharmacological or biological properties [33,34]. Natural products can provide excellent therapeutic effects and safety due to their unique molecular properties [35]. They play an irreplaceable role in drug development. Curcumin can prevent and treat various types of cancers, such as glioblastoma, prostate cancer, breast cancer, and HNC, by inhibiting the PI3K/Akt signaling pathway [36]. 3’-Hydroxypterostilbene effectively inhibits the growth of human colon cancer cells by suppressing the PI3K/Akt signaling pathway [37]. Natural products generally exhibit good biocompatibility, which can reduce side effects. In addition, natural products possess diverse structures, which can offer novel scaffolds or pharmacophores for the development of antitumor drugs.

**Figure 2:**
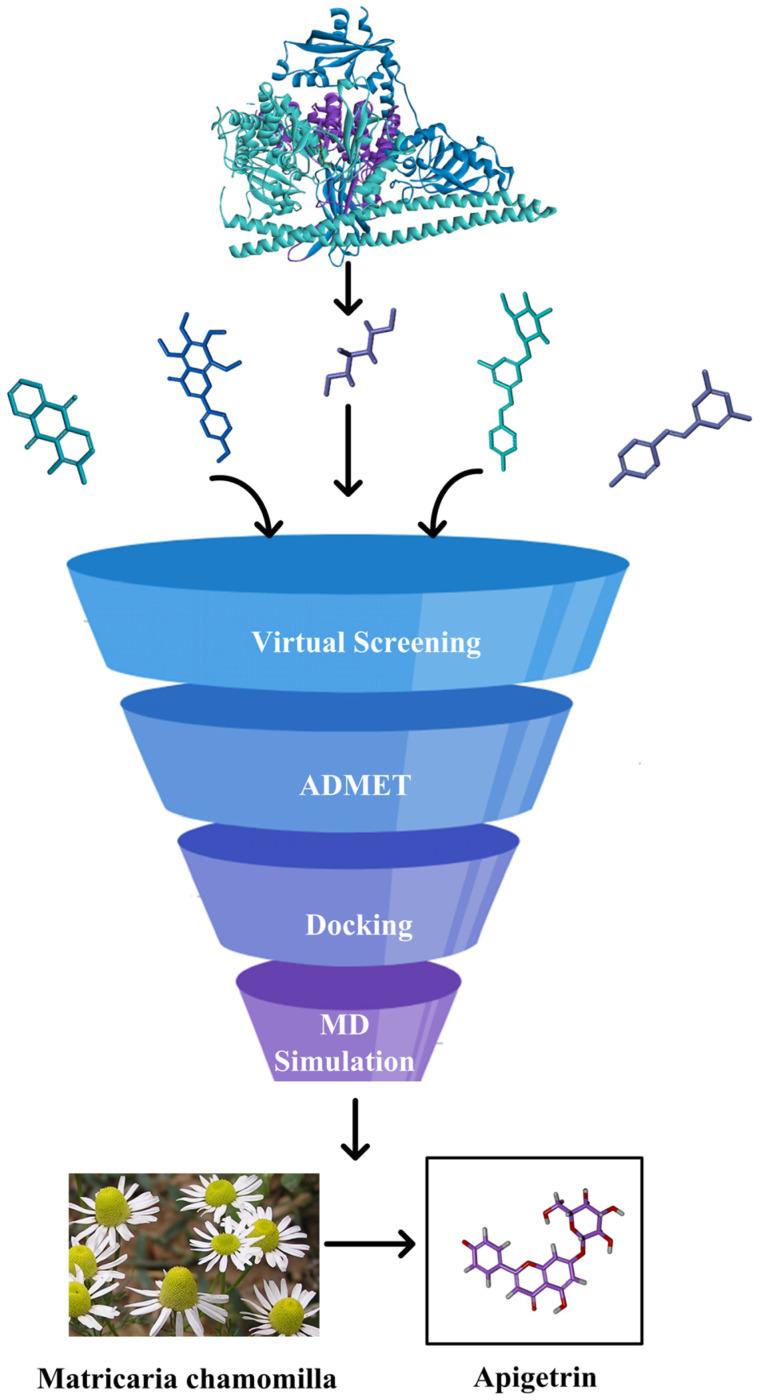
A flow chart showing the identification of the PI3Kα inhibitor.

It is worth further studying whether natural active substances can break the current dilemma of PI3Kα inhibitors. Currently, computer-aided drug design (CADD) technologies, such as high-throughput screening, pharmacophore modeling, scaffold hopping, and ADMET prediction, have been applied to shorten the drug development cycle, reduce research costs, and improve the success rate of drug development. In this study, we first constructed a PI3Kα inhibitor screening model and performed high-throughput virtual screening on a natural product database. The screened compounds were then evaluated for drug-likeness, and, finally, molecular dynamics studies were conducted to reveal the targeting of natural products against PI3Kα (Figure 2).

## 2. Results

### 2.1. Correlation Analysis Between Genes and HNSCC

**Figure 3:**
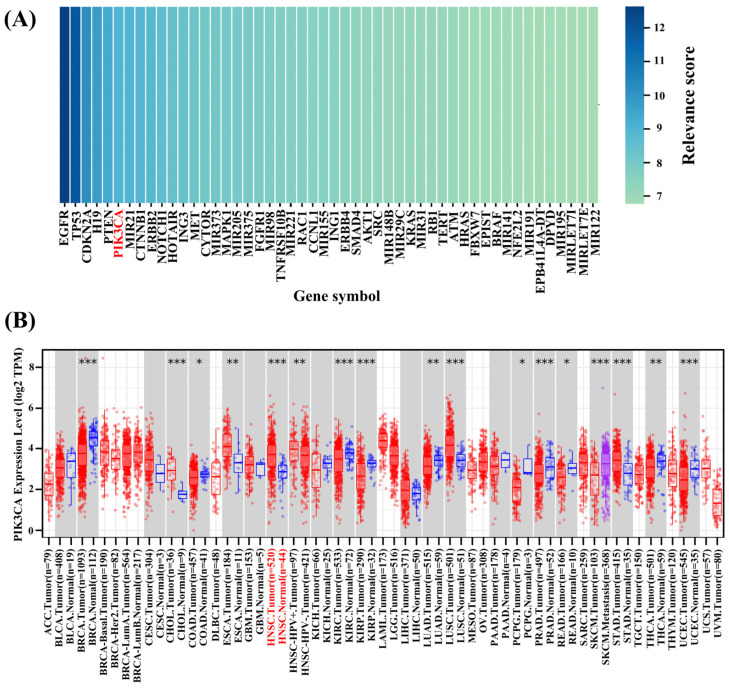
(**A**) Top 50 genes highly associated with HNSCC based on the GeneCards database. The *X*-axis represents gene names, where *PIK3CA* is labeled in red. The *Y*-axis represents the relevance score. The higher the relevance score, the stronger the correlation between the gene and the disease. (**B**) The expression of *PIK3CA* in different types of cancer, where red represents the primary tumor group, purple represents the metastatic tumor group, and blue represents the normal control group. The *X*-axis represents the cancer type, where HNSCC is labeled in red, and the *Y*-axis represents the *PIK3CA* expression level. For cancer types with a normal control group, the background color is displayed in gray. * *p* < 0.05, ** *p* < 0.01, and *** *p* < 0.001.

We searched GeneCards for HNSCC keywords and found 2164 genes (maximum relevance score: 12.71, minimum relevance score: 0.13) related to HNSCC. The higher the relevance score, the stronger the correlation between the gene and disease. Using the median score (0.4) as the screening criterion, 1081 genes that were highly correlated with HNSCC were identified. Among them, *PIK3CA* had a relevance score of 9.02, which was significantly higher than the median and was the sixth-largest gene affecting disease progression in HNSCC patients (Figure 3A).

In addition, we analyzed *PIK3CA* expression in pan-cancer using TIMER 2.0. It could be seen that *PIK3CA* was expressed in multiple types of tumor tissues (Figure 3B). Transcripts Per Million (TPM) can be used to analyze the expression of different genes in cancer and adjacent normal tissues. In Figure 3B, it can be seen that the expression levels of *PIK3CA* in HNSCC, KIRC, KIRP, CHOL, BRCA, STAD, UCEC, SKCM, PRAD, and LUSC were significantly higher than those in normal tissues (*p* < 0.001).

### 2.2. Virtual Database Screening

This section evaluates the binding affinity. In this section, we first constructed a screening model, and the redocking procedure was performed to evaluate the docking reliability. At present, only two PI3Kα inhibitors (Alpelisib and Inavolisib) are approved by the FDA; most drugs are in clinical research, such as Serabelisib and CH5132799, and have significant therapeutic effects. To better evaluate the screening results, we selected multiple PI3Kα inhibitors (Alpelisib, CH5132799, and Serabelisib) for docking with PI3Kα. After docking validation, natural products, Alpelisib, CH5132799, and Serabelisib were docked into the active binding site of PI3Kα. The binding energies of the screened natural products are presented in Table S1. Finally, five molecules with low binding affinities and favorable binding modes were screened. The binding energies of Gallocatechin galleate, Isoacteoside, Apigetrin, Genistin, Rhoifolin, Serabelisib, CH5132799, and Alpelisib were −7.5, −7.3, −7.1, −6.9, −6.3, −6.6, −6.1, and −6.1 kcal/mol, respectively. Detailed information on the five compounds is shown in Table S1.

### 2.3. The Evaluation of the Druggability

**Figure 4:**
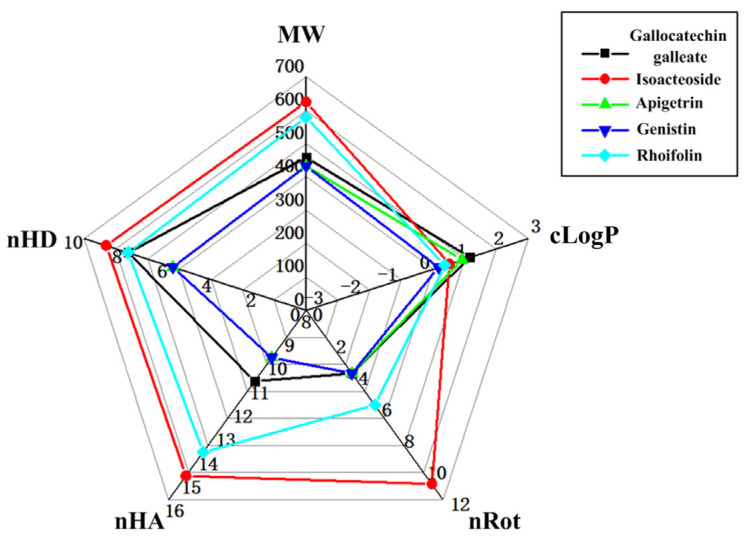
The analysis of Lipinski’s rule of five using a radar chart for molecules.

In this study, we investigated the druggability of the molecules. Lipinski’s rule, including MW, nHD, nHA, nRot, and LogP, is shown in the radar chart in Figure 4 and Table S2. From the results, we could see that Apigetrin and Genistin followed Lipinski’s rule of five. Drugs with high protein binding capacity may have a low therapeutic index. A PPB of <90% is optimal. The PPB of Apigetrin was 83.8%, indicating a favorable therapeutic index. The PPB of Alpelisib was 97.8%, which was higher than 90%, indicating that the therapeutic index of Alpelisib was not as high as that of Apigetrin (Table 1). A plasma clearance rate (CL_plasma_) of less than 5 mL/min/kg indicates a low drug clearance rate. The CL_plasma_ of Apigetrin and Alpelisib were 3.214 and 4.278 mL/min/kg, respectively, indicating low clearance (Table 1). The t_1/2_ of Apigetrin was 3.454 h, indicating that it had a short half-life (Table 1). The t_1/2_ of Alpelisib was 0.815 h, indicating that it was an ultrashort half-life drug (Table 1). In addition, the bioavailability score and Log *K*p of Apigetrin were 0.55 and −7.65 cm/s (Table 1). The bioavailability score and Log *K*p of Alpelisib were 0.55 and −6.71 cm/s (Table 1). The VDss values of Apigetrin and Alpelisib were 0.897 and 1.335 L/kg, respectively, indicating that Apigetrin was primarily distributed in the blood with limited penetration into tissues (Table 1). Apigetrin and Alpelisib had no inhibitory effect on cytochrome P450 (CYP450) enzymes, such as CYP2D6, CYP1A2, and CYP2C9 (Table 1). The probability of Apigetrin and Alpelisib acting as P-gp inhibitors was also relatively low (Table 1).

**Figure 5:**
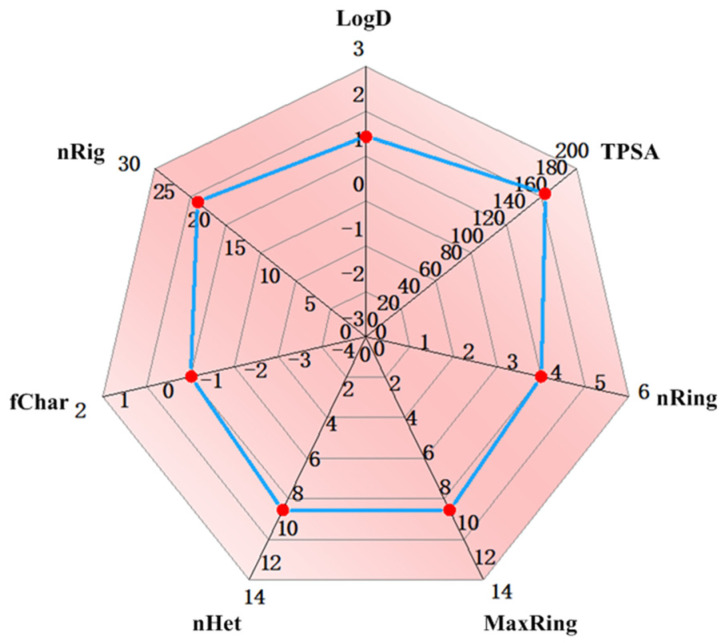
Analysis of physicochemical properties by ADMET lab 3.0.

LD_50_ values exceeding 2000 mg/kg indicate minimal toxicity, while values below 500 mg/kg suggest significant toxicity. Apigetrin exhibited a favorable safety profile, with an estimated LD_50_ of 5000 mg/kg, indicating low acute toxicity (Table 2). In the toxicity assessment, the hepatotoxicity, neurotoxicity, carcinogenicity, immunotoxicity, and cytotoxicity of Apigetrin were predicted to be inactive, with probabilities of 82%, 88%, 86%, 93%, and 69%, respectively (Table 2). However, Alpelisib might have neurotoxicity, with a probability of 74% (Table 2). Additionally, the probabilities of Apigetrin causing acute toxicity in rats, cardiotoxicity (hERG blockers), drug-induced nephrotoxicity, respiratory toxicity, and hematotoxicity were lower than those of Alpelisib (Table 3). We further evaluated the drug-like properties of Apigetrin using ADMET lab 3.0, including nRig, nHet, and so on (Figure 5). From the results, it could be seen that the physicochemical properties of Apigetrin meet the druggability requirements.

**Figure 6:**
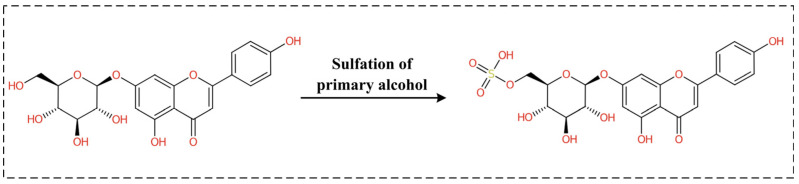
Prediction of the metabolites of Apigetrin.

Next, we used BioTransformer 3.0 to predict the metabolic process of Apigetrin (Figure 6). The metabolic predictions indicated that Apigetrin undergoes sulfation, primarily mediated by sulfotransferase (SULT) enzymes. Apigetrin is a flavonoid compound, and its structure contains a primary alcohol group (-CH_2_OH) on the glucose moiety, which is the main site for the sulfation reaction. During the metabolic process, the primary alcohol group of Apigetrin could react with the sulfate donor 3′-phosphoadenosine-5′-phosphosulfate (PAPS) under the catalysis of SULT, generating a sulfate ester. The metabolite exhibited significantly increased polarity and enhanced water solubility, making it easier to be excreted from the body through urine or bile. Sulfation is an important pathway in phase II metabolism, which helps to convert flavonoids into inactive or low-activity forms, promote their excretion, and reduce potential toxicity.

### 2.4. The Mode of Apigetrin Binding to PI3Kα

**Figure 7:**
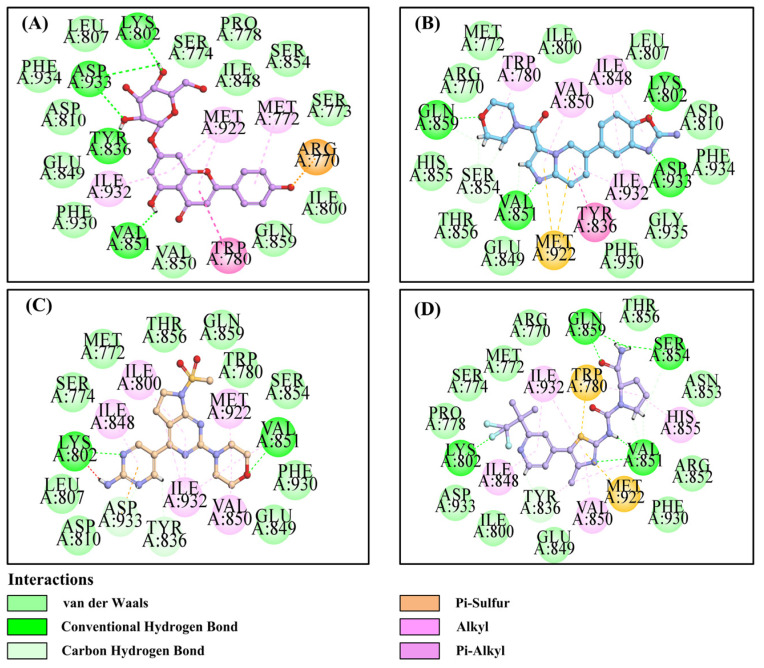
The model of Apigetrin binding to the PI3Kα pocket. (**A**) Two-dimensional (2D) diagrams of Apigetrin–PI3Kα interactions in the PI3Kα pocket; (**B**) 2D diagrams of Serabelisib–PI3Kα interactions in the PI3Kα pocket; (**C**) 2D diagrams of CH5132799-PI3Kα interactions in the PI3Kα pocket; (**D**) 2D diagrams of Alpelisib–PI3Kα interactions in the PI3Kα pocket.

The binding mode of Apigetrin to PI3Kα was generated by Autodock Vina. The results showed that Apigetrin could bind to ATP active pockets, such as Alpelisib, Serabelisib, and CH5132799. The binding modes are shown in Figure 7. Apigetrin could bind to the p110α kinase domain and formed a number of hydrogen bonds (H-bonds) with nearby residues, including Lys802, Tyr836, Val851, and Asp933 (Figure 7A), which generated the same H-bonds as Serabelisib (Lys802, Val851, and Asp933; Figure 7B), CH5132799 (Lys802, and Val851, Figure 7C), and Alpelisib (Lys802 and Val851; Figure 7D). In addition, Arg770 formed Pi interactions with Apigetrin.

### 2.5. Molecular Dynamics Simulation of the PI3Kα–Apigetrin System

**Figure 8:**
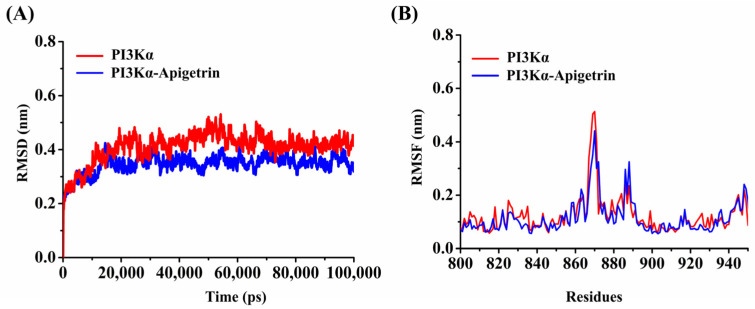
(**A**) RMSD trajectories plot of PI3Kα/the PI3Kα–Apigetrin system during 100,000 ps simulations. (**B**) RMSF trajectories plot of PI3Kα/the PI3Kα–Apigetrin system over the entire simulations.

The Apigetrin–p110α system was subjected to all molecular dynamic simulations, and its binding stability was analyzed. The root mean square deviation (RMSD), which measures the coordinate deviation of a specific atom relative to a reference structure, is often used to evaluate whether a simulation system has reached stability [38]. A stable RMSD means that the corresponding atoms become stable. As shown in Figure 8A, PI3Kα and PI3Kα–Apigetrin reached equilibrium after 20 ns. The RMSD values for the PI3Kα and PI3Kα–Apigetrin systems were 0.413 ± 0.022 nm and 0.343 ± 0.017 nm, respectively, indicating the structural stability and convergence of both systems throughout the simulation.

The root mean square fluctuation (RMSF) calculates the fluctuations in each atom relative to its average position, characterizes the average effect of structural changes over time, and provides a characterization of the flexibility of various regions of the protein [39]. As shown in Figure 8B, the average RMSF values of PI3Kα and PI3Kα–Apigetrin were 0.128 ± 0.005 and 0.115 ± 0.011 nm, respectively. The RMSF value in the PI3Kα–Apigetrin complex system was lower than PI3Kα, indicating Apigetrin could make PI3Kα more stable.

## 3. Discussion

HNSCC has become a serious threat to human health. *PIK3CA* mutations can constitutively activate the PI3K pathway, leading to the occurrence and development of HNSCC [40,41,42]. From certain points of view, activation of the PI3K pathway offers the potential for personalized therapy with PI3Kα inhibitors to improve the treatment outcomes of HNSCC. However, studies of the PI3Kα–inhibitor complex in HNSCC are limited. In this study, we focused on PI3Kα as a promising tumor target for HNSCC therapy.

At present, only two PI3Kα inhibitors have been approved. The reason for this situation is that many drugs fail to achieve the desired results. For example, PI3Kα inhibitors may lead to hyperglycemia in clinical trials, which in turn induces severe hyperinsulinemia. In view of the above, novel agents targeting PI3Kα isoforms are still needed urgently to improve the therapeutic index. In order to find more effective PI3Kα inhibitors for the treatment of HNSCC, we used a computational screening approach to identify potential PI3Kα inhibitors from a database of 12,800 small molecules. The results of the ADMET study showed that Apigetrin had a high druggability. Apigetrin exhibited superior parameters, such as PPB, CL_plasma_, T_1/2_, and VDss, compared to Alpelisib, and it also showed a low inhibitory effect on the CYP450 enzyme family and Pgp. The probability of achieving a bioavailability of greater than 10% in rats was consistent for both Apigetrin and Alpelisib. During hepatic metabolism, Apigetrin can undergo sulfation to generate a sulfate ester metabolite, which has enhanced water solubility and is easily excreted from the body through urine or bile. Apigetrin demonstrated favorable properties in terms of drug absorption, distribution, metabolism, and excretion. Additionally, Apigetrin had low toxicity and a good safety profile. Apigetrin could form H-bonds with the key amino acids Lys802 and Val851 within the active pocket, enabling it to stably bind to the p110α kinase domain. Its binding affinity was superior to that of the reported PI3Kα inhibitor, Alpelisib. MD simulations were used to evaluate the stability of the Apigetrin–PI3Kα complex. The results showed that Apigetrin could stably bind to PI3Kα.

Structure-based virtual screening relies on the three-dimensional structural information of the target protein to simulate and predict the interactions between compounds and the target protein, enabling the rapid screening of potential active molecules from large compound libraries. This technology has advantages such as high efficiency and low costs in drug development. The computational results indicated that Apigetrin could stably bind to the ATP active pocket and had the potential to inhibit PI3Kα activity.

However, this technology has certain limitations, and it cannot fully reflect an authentic biological environment. Therefore, it is necessary to further validate the results through in vitro and in vivo experiments. For instance, kinase assays can be employed to investigate the inhibitory effects of compounds on PI3Kα, while WST-8 assays can be utilized to detect the impact of Apigetrin on the proliferation inhibition of HNSCC cells. Further in vitro and in vivo investigations are warranted to validate the anti-HNSCC efficacy and elucidate the precise molecular mechanisms of Apigetrin.

## 4. Materials and Methods

### 4.1. Correlation Analysis Between PIK3CA and HNSCC

We searched for HNSCC keywords in the GeneCards database (https://www.genecards.org/) to identify genes related to HNSCC [43]. We calculated the median of the relevance score and selected genes. The data were analyzed to determine highly correlated genes. The expression of *PIK3CA* in pan-cancer was analyzed using the TIMER 2.0 database [44].

### 4.2. Virtual Screening Study

By searching the Protein Data Bank (https://www.rcsb.org/), we download the three-dimensional structure of PI3Kα as a PDB file (ID: 4JPS). Firstly, we prepared the protein, including removing water molecules and solvent molecules from the protein, adding hydrogen atoms, and then converting the protein file to pdbqt format by Autodock Vina [45,46,47]. The x, y, and z of the cubic grid box were 45, 47, and 61, respectively. The docking algorithm was validated by redocking the co-crystallized ligands. The computed RMSD between the experimental and docked poses was found within the threshold limit (<2 Å). A total of 12,800 molecules with a molecular weight not exceeding 700 Da and possessing biological activity were collected from the natural product activity and species source database (NPASS, Pubchem) and the flavonoid compound database (Selleckchem). Then, we prepared and converted the ligand file to pdbqt format before virtual screening. Then, the molecules were screened using Autodock Vina v1.2.0. The conformation with the highest binding energy and lowest RMSD was considered to be the optimal conformation. In addition to comparing the binding energies with the existing original ligand Alpelisib in 4JPS, we also selected the PI3Kα inhibitors Serabelisib and CH5132799 for comparison. Finally, natural products with binding energies higher than these PI3Kα inhibitors were selected for further evaluation.

The Discovery studio visualizer (BIOVIA Discovery studio 2024 Client, https://discover.3ds.com/discovery-studio-visualizer-download, accessed on 6 July 2024) was used to study the interactions between proteins and ligands and to analyze the number and types of bonds, such as H-bonds, hydrophobic interactions, and van der Waals forces.

### 4.3. Lipinski’s Filter and ADMET Study

This section used ADMET lab 3.0 (https://admetmesh.scbdd.com) and SwissADME (http://www.swissadme.ch/, accessed on 26 December 2024) to evaluate the Lipinski’s properties and pharmacokinetic parameters of molecules [48]. The contents of the evaluation were as follows: molecular weight (MW), number of rotatable bonds (nRot), number of hydrogen bond acceptors (nHA), number of hydrogen bond donors (nHD), cLogP, PPB, BBB, Bioavailability score, Log Kp, and so on. The ProTox-3.0 platform was used to evaluate the potential toxicity of Apigetrin, including hepatotoxicity, neurotoxicity, carcinogenicity, and so on [49]. Finally, the physicochemical properties of Apigetrin, including LogD, nRing, MaxR, TPSA, etc., were re-verified using ADMET lab 3.0 (https://admetmesh.scbdd.com) [50]. The metabolic pathways and metabolites of Apigetrin were analyzed using BioTransformer 3.0.

### 4.4. Molecular Dynamics Simulation

The molecular dynamics (MD) simulations were carried out by GROMACS 2020.3 software. The amber99sb-ildn force field and the general Amber force field (GAFF) were used to generate the parameters and topologies of the proteins and ligands, respectively. The operational steps were as follows. (1) The simulation box size was optimized with the distance between each atom of the protein and the box being greater than 1.0 nm. (2) The box was filled with water molecules based on a density of 1. (3) The water molecules were replaced with Cl^−^ and Na^+^ ions to make the simulation system electrically neutral. (4) The unreasonable contact or atom overlap in the entire system was reduced by the steepest descent method, where energy optimization with 5.0 × 10^4^ steps was performed to minimize the energy consumption of the entire system. (5) After energy minimization, first-phase equilibration was performed using the NVT ensemble at 300 K for 100 ps to stabilize the temperature of the system. Second-phase equilibration was simulated with the NPT ensemble at 1 bar and 100 ps. (6) MD simulations were performed for 100 ns and repeated three times. The system was run with 300 K and 1 atmosphere [4].

## Figures and Tables

**Table 1:** The ADME properties of Apigetrin and Alpelisib.

	Apigetrin	Alpelisib
PPB ^1^ (%)	83.8%	97.8%
BBB ^2^ (%)	No	No
CL_plasma_^3^ (mL/min/kg)	3.214	4.278
T_1/2_^4^ (h)	3.454	0.815
CYP2D6 inhibitor ^5^	No	No
Bioavailability score ^6^	0.55	0.55
Log *K*p ^7^ (cm/s)	−7.65	−6.71
VDss ^8^ (L/kg)	0.897	1.335
CYP1A2 inhibitor ^9^	No	No
CYP2C9 inhibitor ^10^	No	No
CYP3A4 inhibitor ^11^	No	Yes
Pgp inhibitor (%) ^12^	0–10%	0–10%

1. PPB: plasma protein binding; optimal: <90%. 2. BBB: blood–brain barrier penetration. 3. CL_plasma_: plasma clearance rate; >15 mL/min/kg: high clearance; 5–15 mL/min/kg: moderate clearance; <5 mL/min/kg: low clearance. 4. T_1/2_: ultrashort half-life drugs: T_1/2_ < 1 h; short half-life drugs: T_1/2_ between 1 and 4 h; intermediate short half-life drugs: T_1/2_ between 4 and 8 h; long half-life drugs: T_1/2_ > 8 h. 5. CYP2D6: ‘no’ represents no inhibitory effect on the enzyme. 6. Bioavailability score: probability of F > 10% in rats. 7. Log *K*p: skin permeation value. 8. VDss: volume distribution; optimal: 0.04–20 L/kg. 9. CYP1A2 inhibitor: ‘no’ represents no inhibitory effect on the enzyme. 10. CYP2C9 inhibitor: ‘no’ represents no inhibitory effect on the enzyme. 11. CYP3A4 inhibitor: ‘no’ represents no inhibitory effect on the enzyme; ‘yes’ represents an inhibitory effect on the enzyme. 12. Pgp inhibitor: the probability of being a Pgp inhibitor.

**Table 2:** The toxicity assessment results of Apigetrin and Alpelisib by ProTox-3.0.

	Apigetrin	Alpelisib
LD_50_ (mg/kg)	5000	1000
Hepatotoxicity	Inact82%	Inact54%
Neurotoxicity	Inact88%	Act74%
Carcinogenicity	Inact86%	Inact58%
Immunotoxicity	Inact93%	Inact97%
Cytotoxicity	Inact69%	Inact68%

Inact%: the probability of predicting that the compound is non-toxic. Act%: The probability of predicting that the compound is toxic.

**Table 3:** The toxicity assessment results of the Apigetrin and Alpelisib by ADMET 3.0.

	Apigetrin	Alpelisib
Rat oral acute toxicity	4.7%	44.4%
hERG blockers	2.8%	43.5%
Drug-induced nephrotoxicity	34.7%	88.5%
Respiratory toxicity	3.6%	68.1%
Hematotoxicity	12.5%	66.1%

The data in the table represent the probability of toxicity.

## Data Availability

The original contributions presented in this study are included in this article and Supplementary Materials; further inquiries can be directed to the corresponding authors.

## Supplementary Materials

The supporting information can be downloaded at https://www.mdpi.com/article/10.3390/ijms26083565/s1.
