# Supplementary material for: In Silico Discovery of a Novel Natural Product Targeting PI3Kα for the Treatment of Head and Neck Squamous Cell Carcinoma
Source: Int J Mol Sci. 2025 Apr 10;26(8):3565. doi: 10.3390/ijms26083565 (PMC12027195; doi:10.3390/ijms26083565)
Supplement: Supplementary file 1 [file ijms-26-03565-s001.zip › Table S1.pdf]

Table S1. The structures and binding energy of molecules.

ID number	Structure	Binding energy (kcal/mol)
Gallocatechin galleate	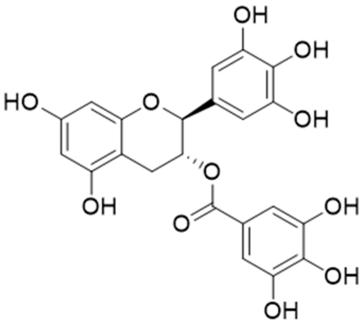	-7.5
Isoacteoside	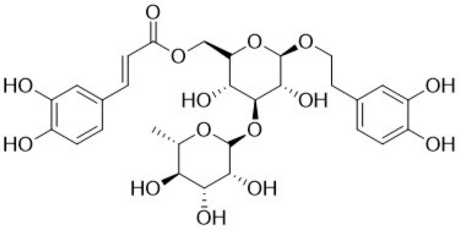	-7.3
Apigetrin	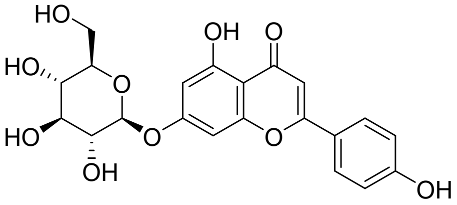	-7.1
Genistin	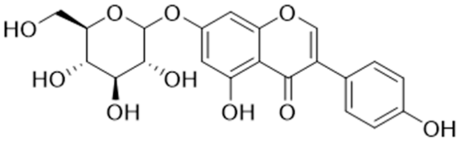	-6.9
Rhoifolin	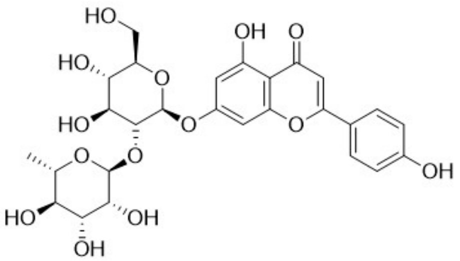	-6.3
Serabelisib	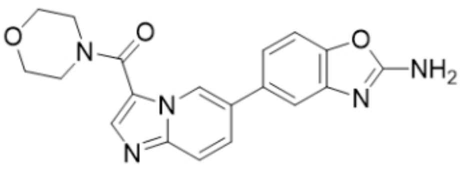	-6.6

CH5132799	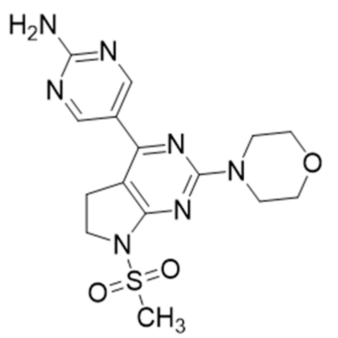	-6.1
Alpelisib	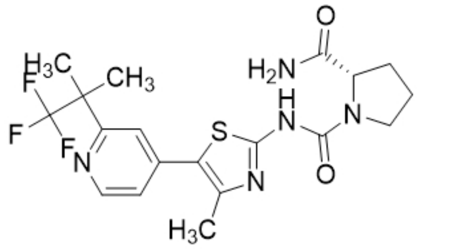	-6.1
