# Supplementary material for: In Silico Discovery of a Novel Natural Product Targeting PI3Kα for the Treatment of Head and Neck Squamous Cell Carcinoma
Source: Int J Mol Sci. 2025 Apr 10;26(8):3565. doi: 10.3390/ijms26083565 (PMC12027195; doi:10.3390/ijms26083565)
Supplement: Supplementary file 1 [file ijms-26-03565-s001.zip › Table S2.pdf]

Table S2. The Lipinski's filter of the top 5 compounds with docking score.

ID number	MW[1]	LogP[2]	nRot[3]	nHA[4]	nHD[5]
**Gallocatechin galleate**	458.37	1.481	4	11	8
**Isoacteoside**	624.59	0.872	11	15	9
**Apigetrin**	432.11	1.213	4	10	6
**Genistin**	432.38	0.579	4	10	6
**Rhoifolin**	578.52	0.749	6	14	8

1. MW: 0~500; **2.** LogP: ≤5; **3.** nRot: 0~10; **4.** nHA: 0~10; **5.** nHD: 0~5.
